# Mediterranean Diet, Sleep Quality, Perceived Stress, and Physical Activity: A Pilot Study Among School Teachers

**DOI:** 10.3390/nu17172745

**Published:** 2025-08-25

**Authors:** Marta Esgalhado, António Raposo, Najla A. Albaridi, Thamer Alslamah, Nada Alqarawi, Leandro Oliveira

**Affiliations:** 1 CBIOS (Research Center for Biosciences and Health Technologies), ECTS (School of Health Sciences and Technologies), Lusófona University, Campo Grande 376, 1749-024 Lisboa, Portugal; marta.esgalhado@ulusofona.pt; 2 Department of Health Science, College of Health and Rehabilitation, Princess Nourah bint Abdulrahman University, P.O. Box 84428, Riyadh 11671, Saudi Arabia; naalbaridi@pnu.edu.sa; 3 Department of Public Health, College of Applied Medical Sciences, Qassim University, Buraydah 51452, Saudi Arabia; 4037@qu.edu.sa; 4 Department of Psychiatric and Mental Health, and Community Health, College of Nursing, Qassim University, Buraydah 51452, Saudi Arabia; n.alqarawi@qu.edu.sa

**Keywords:** Mediterranean diet, sleep quality, school teachers, PSQI, MEDAS, lifestyle, stress

## Abstract

**Background:** Lifestyle behaviours, such as dietary patterns, sleep quality, perceived stress, and physical activity, are closely interconnected and play a critical role in maintaining health and well-being. Among school teachers, a profession marked by high psychosocial demands, the interplay between these factors warrants further exploration. **Objective:** This pilot study aimed to explore the associations between adherence to the Mediterranean Diet (MD), sleep quality, perceived stress, and physical activity levels among Portuguese primary and secondary school teachers. **Methods:** A cross-sectional study was conducted between April and December 2023. Participants completed validated self-reported instruments, including the MD Adherence Screener (MEDAS), the Pittsburgh Sleep Quality Index (PSQI), the Perceived Stress Scale (PSS-10), and the short form of the International Physical Activity Questionnaire (IPAQ-SF). Correlational analyses and multivariable linear regression models were applied to explore the relationships among the variables. **Results:** Among the 50 teachers, 32% demonstrated high adherence to the MD, 60% reported good sleep quality, 62% experienced moderate stress, and 44% engaged in high physical activity. Women were more likely to adhere strongly to the MD (*p* = 0.012). Higher MD adherence was positively associated with physical activity (ρ = 0.343; *p* = 0.015). A positive correlation was observed between perceived stress and poorer sleep quality (ρ = 0.346, *p* = 0.014), and a negative correlation between perceived stress and physical activity levels (ρ = −0.297, *p* = 0.036). Despite reporting good sleep quality, these participants had higher perceived stress scores (*p* = 0.015). In adjusted models, sleep quality was the only significant predictor of perceived stress (B = 0.708; *p* = 0.003), and vice versa (B = 0.267; *p* = 0.003), suggesting a bidirectional relationship. **Conclusions:** The findings highlight the interrelation between dietary pattern, sleep stress, and physical activity in a professional group vulnerable to lifestyle-related health challenges. Although the sample size limits generalisability, this study provides preliminary evidence supporting the need for integrated health promotion strategies targeting stress reduction and lifestyle optimisation among educators.

## 1. Introduction

In recent decades, the growing prevalence of non-communicable chronic diseases, such as cardiovascular diseases, type 2 diabetes, obesity, depression, and sleep disorders, has brought healthy lifestyle behaviours to the forefront of public health concerns [1,2]. Within this context, the Mediterranean Diet (MD) has emerged as an exemplary dietary pattern [3]. It is valued not only for its rich nutritional profile but for its association with a broad range of physical, mental, and emotional health benefits [4,5]. Beyond nutrition, other lifestyle factors, including sleep quality, perceived stress, and regular physical activity, have been identified as essential and interrelated determinants that significantly influence overall well-being [6]. Considering these factors together allows for a more holistic and realistic understanding of human health, one that takes into account the complex interactions between behaviour, biology, and environment [7,8].

The MD, recognised by UNESCO as an Intangible Cultural Heritage of Humanity, is rooted in the traditional dietary habits of countries surrounding the Mediterranean Sea, including Portugal [9]. This dietary pattern is characterised by a high intake of plant-based foods (e.g., fruits, vegetables, legumes, nuts, and whole grains), with olive oil as the main source of fat. It also includes the moderate consumption of fish, poultry, and dairy products, and the low consumption of red meat and processed foods [10]. Importantly, the MD also embraces the principles of social and sustainable eating, seasonality, and physical activity as part of a broader lifestyle philosophy [11]. Numerous scientific studies have shown that adherence to this pattern is associated with a lower risk of metabolic, inflammatory, and neurodegenerative diseases, as well as improvements in mood, sleep quality, and cognitive performance [4,12,13,14].

The quality of an individual’s diet can directly affect the gut–brain axis, systemic inflammation, gut microbiota composition, and the production of neurotransmitters [15], all of which modulate sleep, stress, and mood regulation [16]. A diet rich in anti-inflammatory and antioxidant nutrients, such as the MD, may support the regulation of circadian rhythms and promote better sleep quality [17]. Individuals adhering to such patterns often report lower levels of stress, anxiety, and fatigue [18,19]. This suggests that nutrition may serve as a modifiable factor in emotional regulation and stress resilience [20].

Conversely, poor or insufficient sleep has been linked to cognitive impairment, hormonal imbalances, increased risk of cardiovascular disease, greater appetite, and a preference to consume energy-dense, nutrient-poor foods [19]. Chronic sleep deprivation is frequently associated with elevated perceived stress, emotional instability, and impaired coping abilities [21]. On the other hand, good sleep quality appears to protect against the adverse effects of stress, acting as a critical mechanism for physical and emotional recovery [22].

Perceived stress, particularly when chronic, plays a critical role in occupational health and lifestyle, having been associated with immune dysregulation, greater vulnerability to disease, and disturbances in both eating and sleeping patterns [23,24]. The stress response is shaped by several factors, including personal resilience, social support networks, and occupational context [25]. In this regard, regular physical activity is a protective factor that contributes not only to maintaining a healthy weight and improving metabolic indicators, but to reducing the symptoms of anxiety, stress, and depression [26,27]. Physical exercise enhances the release of endorphins and supports emotional regulation, strengthening an individual’s ability to adapt to challenging situations [28].

The relationship between diet, sleep, stress, and physical activity is well established, with research consistently showing that these factors are closely interconnected. For example, healthy dietary habits can improve sleep patterns [29] and can help lower stress levels [30]. In turn, better sleep can support healthier dietary choices [31], and lower stress levels are associated with increased motivation to engage in physical activity [32]. Likewise, active individuals tend to report lower stress levels and a better sleep quality [23]. These dynamics are particularly important in professional environments that demand emotional, cognitive, and physical resilience, such as the education sector [33].

Teachers, especially those working in primary and secondary education, often face high levels of occupational stress [34]. Their professional duties involve heavy workloads, constant pedagogical and emotional demands, the need for continuous professional development, and the daily challenges of managing diverse and dynamic classrooms [35]. These factors contribute to physical and mental exhaustion, which may lead to difficulties maintaining healthy lifestyle routines, including consistent dietary habits, regular physical activity, and adequate sleep [36]. These challenges can be further exacerbated by personal circumstances (e.g., age, family responsibilities), and systemic pressures within the educational system. It is therefore crucial to understand the lifestyle behaviours of teachers from an integrated perspective and to identify potential pathways for improving their overall well-being [37].

In Portugal, although awareness of teacher health and well-being is increasingly recognised, to the best of our knowledge, no studies have yet explored the combined relationship between adherence to the MD, sleep quality, perceived stress, and physical activity in the teaching population. The existing research tends to focus on isolated variables, without addressing the complex and dynamic interactions among these key dimensions of health [38,39,40]. Moreover, the available data are often outdated, limited in scope, or not representative of the Portuguese educational context [38,39,40]. This significant gap in the literature hinders the development of effective, evidence-based interventions that are tailored to the specific needs and challenges faced by teachers.

Primary and secondary school teachers represent a professional group with a significant impact on both the educational and the public health systems [41]. Due to their role in shaping students’ behaviours, values, and daily routines, teachers act as health role models within the school community [42]. However, they are also frequently exposed to chronic work-related stress, emotional exhaustion, and time constraints, which may compromise their own health behaviours and increase their vulnerability to non-communicable diseases [34,43]. As such, improving the health and well-being of teachers is not only essential from an occupational health perspective, but has broader public health implications [44]. Promoting teachers’ well-being can lead to more positive classroom climates, increased motivation and retention among staff, and improved student outcomes [37]. By targeting teachers in health promotion efforts, it is possible to foster a healthier school environment and influence the health trajectories of future generations through positive modelling [33,41].

Given these considerations, exploratory research is essential to characterise the lifestyle behaviours of Portuguese school teachers, identify the relevant patterns and associations among core health variables, and inform the design of holistic, targeted strategies to improve well-being in educational settings [45].

This pilot study aims to analyse the associations between adherence to the MD, sleep quality, perceived stress, and physical activity levels among Portuguese primary and secondary school teachers. By identifying behavioural patterns and interactions among these variables, this study seeks to enhance our understanding of teachers’ lifestyles and to provide a foundation for developing more effective and context-specific health promotion strategies within the educational sector.

## 2. Materials and Methods

### 2.1. Study Design and Population

This descriptive, cross-sectional study was approved by the Ethics Committee of the School of Health Sciences and Technologies of Universidade Lusófona (approval no. P04-23), on 31 March 2023.

Inclusion criteria included (a) active primary or secondary school teachers working in Portugal, (b) aged ≥18 years, and (c) consenting to participate. Exclusion criteria included (a) not currently teaching, (b) incomplete responses, and (c) refusal to consent.

**Figure 1:**
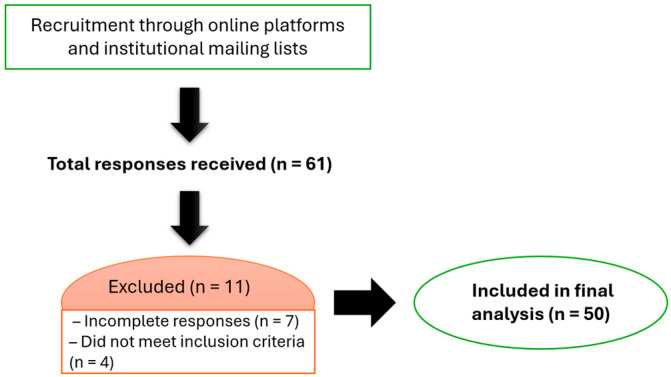
Flow diagram illustrating the recruitment, eligibility screening, and final sample size.

From an initial pool of 61 responses, 11 were excluded, 7 due to incomplete data and 4 for not meeting the inclusion criteria, resulting in a final analytical sample of 50 participants. This process is summarised in Figure 1.

Prior to participation, all individuals were informed about the objectives and procedures of this study and provided their informed digital consent. Participation was entirely voluntary, and respondents were assured of both their anonymity and the confidentiality of their responses. No personally identifiable data was collected at any stage. All responses were gathered anonymously and securely stored on an encrypted institutional server. Participants retained the right to discontinue the questionnaire at any time without any consequences. This study used a convenience sampling method. Data collection took place between April and December 2023, using a self-administered online questionnaire, estimated to require approximately 15 min to complete. The survey was disseminated through digital platforms (WhatsApp^®^, Facebook^®^, Instagram^®^), institutional mailing lists, and personal networks. The dissemination was supported by schools and professional associations with which the research team maintained academic and institutional affiliations.

Eligibility was verified through an initial screening question confirming the respondent’s current teaching profession. Additional questions regarding type of school, teaching level, and years of professional experience were included to ensure internal consistency.

### 2.2. Questionnaire and Survey Instruments

The questionnaire was composed exclusively of validated instruments adapted for the Portuguese population, namely the Mediterranean Diet Adherence Screener (MEDAS), the Pittsburgh Sleep Quality Index (PSQI), the 10-item Perceived Stress Scale (PSS-10), and the International Physical Activity Questionnaire-Short Form (IPAQ-SF). No custom-developed items or unvalidated measures were used.

The questionnaire, written in Portuguese, was structured into five sections covering sociodemographic and professional characteristics, dietary habits, sleep quality, perceived stress, and physical activity. The first section collected information on biological sex, age, region of residence (classified according to NUTS II), education attainment, teaching level (primary or secondary), years of teaching experience, tobacco use, and self-reported weight and height. Body mass index (BMI) was calculated using the standard formula (weight in kilograms divided by height in meters squared), and nutritional status was classified according to the World Health Organization guidelines [46].

Adherence to the MD was assessed using the 14-item MEDAS, a validated instrument for the Portuguese population [47]. Each item is scored dichotomously (yes = 1, no = 0), yielding a total score ranging from 0 to 14. Higher scores indicate greater adherence to the Mediterranean dietary pattern [48]. For analytical purposes, adherence was categorised as low (≤5 points), moderate (6–9 points), or high (≥10 points), based on the cut-offs established in the literature [49].

Sleep quality was measured using the PSQI, a validated tool for the Portuguese population [50,51]. The PSQI consists of 19 items, grouped into seven components, including subjective sleep quality, sleep latency, sleep duration, habitual sleep efficiency, sleep disturbances, use of sleep medication, and daytime dysfunction. Each component is scored from 0 to 3, yielding a global score ranging from 0 to 21. A global score greater than 5 indicates clinically significant poor sleep quality [50].

Perceived stress was assessed using the 10-item version of the PSS-10 [52], translated and validated for the Portuguese population [53]. The PSS evaluates perceived unpredictability, uncontrollability, and overload experienced over the past few weeks. Items are rated on a five-point Likert scale (0 = never to 4 = very often), resulting in a total score ranging from 0 to 40, with higher scores indicating greater levels of perceived stress.

Physical activity was assessed using the short form of IPAQ-SF, validated for use in the Portuguese population [54]. The questionnaire gathers information on the frequency and duration of physical activities over the previous seven days, including vigorous and moderate activity, walking, and sedentary time [55]. According to the IPAQ scoring guidelines, the participants were categorised as having low, moderate, or high levels of physical activity [54].

A complete version of the online questionnaire, including all instruments in their validated Portuguese format, is provided in the Supplementary Materials.

### 2.3. Statistical Analysis

All statistical analyses were conducted using Jamovi software (version 2.4.14; the jamovi project, www.jamovi.org). Continuous variables were tested for normality using the Shapiro–Wilk test. Normally distributed variables were expressed as mean and standard deviation, whereas non-normally distributed variables were summarised as median and interquartile range. Categorical variables were presented as absolute and relative frequencies.

Group comparisons for categorical variables were performed using the chi-squared test or Fisher’s exact test, as appropriate. For continuous variables, independent samples *t*-tests were used for normally distributed data, and the Mann–Whitney U test (for two groups) or the Kruskal–Wallis test (for more than two groups) for non-parametric comparisons. Spearman’s rank correlations were calculated to examine associations between the global PSQI score and its component scores. Regression analyses were conducted to explore the interrelationships among MD adherence (MEDAS score), sleep quality (PSQI score), perceived stress (PSS-10), and physical activity levels (categorised as high versus low). Four regression models were estimated, each including one of the variables as the dependent outcome and the others as predictors. All models were adjusted for sex, age, and teaching level. When performing multiple comparisons across the 14 MEDAS items, the Bonferroni correction was applied to account for an inflated type I error. Accordingly, statistical significance was adjusted to *p* < 0.0036 (0.05/14). For all other analyses, statistical significance was set at *p* < 0.05 (two-tailed).

## 3. Results

### 3.1. General Characteristics

A total of 50 school teachers participated in this study, the majority of whom were female (82%), as detailed in Table 1. The median age of the sample was 53 years. Most participants resided in the Lisbon Metropolitan Area (68%) and held at least a bachelor’s degree (74%). The median duration of teaching experience was 26 years, with the highest proportion employed at the high school level (48%).

Regarding health-related characteristics, 58% of the participants had a BMI within the normal weight range, with a median of 23 kg/m^2^. Furthermore, the majority were non-smokers (76%). No statistically significant differences were observed between the sexes across the variables analysed.

### 3.2. Adherence to the Mediterranean Dietary Pattern

Table 2 presents the distribution of responses to the 14-item MEDAS (stratified by sex. Most teachers reported using olive oil as their main culinary fat (98%), although only 24% met the recommended intake of ≥4 tablespoons per day. More than 70% of the participants reported low consumption of butter, margarine, or cream; sugary or carbonated beverages; commercial baked goods and pastries; and preference for white meat over red meat. A similarly high percentage indicated frequent consumption of dishes prepared with a Mediterranean-style sautéed base (tomato, onion, garlic, and olive oil).

Female participants showed higher adherence to recommendations regarding the intake of vegetables, nuts, white meat, and Mediterranean-style sautéed dishes, as well as a lower consumption of red meat. By contrast, male participants reported higher consumption of pulses, fruit, fish or seafood, and wine, and a greater proportion of men also reported low consumption of butter, margarine, or cream, and sugary or carbonated beverages. Among the 14 items assessed, only the frequency of consuming Mediterranean-style sautéed dishes showed a nominally significant difference between the sexes (*p* = 0.035). However, when applying the Bonferroni correction to account for multiple comparisons (adjusted significance threshold α = 0.0033), this difference no longer reached statistical significance. This adjustment mitigates the risk of type I errors, underscoring the importance of a cautious interpretation of isolated *p*-values in the context of multiple testing.

The overall MEDAS score, reflecting global adherence to the Mediterranean Diet, was similar between men and women (median = 9), indicating moderate adherence with no statistically significant differences observed.

Supplementary Materials Table S1 presents the sociodemographic, professional, and health-related characteristics of the participants, stratified by the level of adherence to the MD (low, moderate, high). Higher adherence was significantly more prevalent among female participants (*p* = 0.012). No other statistically significant differences were observed across adherence levels.

### 3.3. Sleep Quality

Supplementary Materials Table S2 displays the distribution of the PSQI components according to levels of adherence to the MD. Overall, no statistically significant differences were found between groups in any of the PSQI components or in the global sleep quality score. A majority of the participants reported good subjective sleep quality and an average sleep duration of 6 to 7 h per night. Sleep latency, efficiency, daytime disturbances, and sleep medication use were similarly distributed among the adherence groups, with no discernible trend. The median global PSQI scores were comparable across all the adherence categories, indicating no association between MD adherence and overall sleep quality in this sample.

Supplementary Materials Table S3 presents the correlations between the global PSQI score and each of its components. All components showed statistically significant positive correlations with the global score, indicating that poorer outcomes in individual dimensions are associated with worse overall sleep quality. The strongest associations were observed with sleep medication use (ρ = 0.770, *p* < 0.001), sleep efficiency (ρ = 0.690, *p* < 0.001), and subjective sleep quality (ρ = 0.663, *p* < 0.001). Moderate correlations were found with sleep latency (ρ = 0.609), sleep disturbances (ρ = 0.569), and daytime dysfunction (ρ = 0.429), while sleep duration presented a weaker but still significant association (ρ = 0.404, *p* = 0.004).

Supplementary Materials Table S4 presents the sociodemographic, professional, and health-related characteristics stratified by sleep quality. Although no statistically significant differences were identified, some variations were observed across the groups, which, however, should be interpreted with caution given the lack of statistical support. Participants with good sleep quality had more years of teaching experience. A higher proportion of men were found in the good sleep quality group (20.0% vs. 15.0%), whereas women were more represented among those with poor sleep quality (85.0% vs. 80.0%). Obesity appeared more frequently in the group with poor sleep quality (15.0% vs. 3.3%), and only teachers from primary education were present in the group with good sleep quality. While these variations did not reach statistical significance, they may reflect relevant patterns to be further explored in future research with larger and more diverse samples.

### 3.4. Adherence to the Mediterranean Diet vs. Sleep Quality

Supplementary Materials Table S5 presents the distribution of the PSS-10 responses stratified by adherence to the MD and sleep quality. No statistically significant differences were found across the MD adherence groups, either in individual item responses or the global perceived stress scores. Descriptively, participants with high MD adherence reported lower global PSS-10 scores (14.5) compared to those with low (15.0) and moderate (17.0) adherence.

However, significant associations emerged when the participants were stratified by sleep quality. Unexpectedly, teachers with good sleep quality reported more frequent experiences of being upset by unexpected events (*p* = 0.004) and of feeling nervous or stressed (*p* = 0.016). This counterintuitive pattern suggests that better sleep does not necessarily correspond to lower perceived stress in specific situations. Moreover, the global PSS-10 score was significantly higher among those with good sleep quality (18.0 ± 6.59) compared to those with poor sleep quality (14.3 ± 3.80; *p* = 0.015), indicating that individuals classified as good sleepers reported greater overall perceived stress.

Supplementary Materials Table S6 summarises the participants’ sociodemographic, professional, and health-related characteristics according to their levels of perceived stress. No statistically significant differences were found across the stress groups. Nonetheless, participants with lower stress levels were more frequently non-smokers and had greater adherence to the MD, although these differences were not statistically significant. Importantly, sleep quality, as measured by the PSQI, showed a significant worsening with increasing stress levels (*p* = 0.043), supporting an association between higher stress levels and poorer sleep quality.

Supplementary Materials Table S7 presents the participants’ characteristics according to their levels of physical activity. Although none of the differences reached statistical significance, participants with higher levels of physical activity had higher adherence to the MD (higher MEDAS scores and more frequency in the high adherence group), and reported slightly lower perceived stress, as reflected by lower PSS-10 scores. Moreover, participants with lower physical activity levels presented higher median BMI and a greater proportion of individuals classified as obese. No meaningful differences were observed.

Table 3 revealed several statistically significant associations among the variables analysed. A positive correlation was observed between adherence to the MD and physical activity levels (ρ = 0.343), indicating that individuals with higher dietary adherence were also more likely to engage in higher levels of physical activity. Similarly, a positive correlation emerged between sleep disturbances (PSQI score) and perceived stress (PSS-10 score) (ρ = 0.346), suggesting that higher stress is associated with poorer sleep quality. By contrast, physical activity levels were inversely correlated with perceived stress (ρ = −0.297), implying a potential protective role of regular physical activity against psychological stress. No other significant correlations were identified.

Regression analyses demonstrated a significant bidirectional relationship between perceived stress (PSS-10) and sleep quality (PSQI), as detailed in Table 4. Specifically, higher PSQI scores, indicative of poorer sleep quality, were associated with increased perceived stress levels (B = 0.267; 95% CI: 0.153–0.712; *p* = 0.003). Conversely, greater perceived stress was strongly linked to worse sleep quality (B = 0.708; 95% CI: 0.154–0.718; *p* = 0.003). No other statistically significant associations were identified among MD adherence (MEDAS), sleep quality, perceived stress, and physical activity levels. Nonetheless, there was a non-significant trend suggesting that higher adherence to the MD might be associated with increased physical activity (OR = 0.452; *p* = 0.056).

## 4. Discussion

This pilot study explored the interconnections between adherence to the MD, sleep quality, perceived stress, and physical activity among Portuguese school teachers, a professional group frequently exposed to elevated occupational stress and associated health risks The findings revealed several meaningful behavioural and psychological patterns offering insight into how modifiable lifestyle factors may collectively influence stress regulation and overall well-being.

Consistent with the previous research, female participants exhibited higher adherence to the MD, corroborating evidence that women are more likely than men to engage in health-promoting dietary behaviours [56,57]. This sex-based disparity is often attributed to the greater nutritional awareness, higher health literacy, and sociocultural norms that influence food choices [58,59]. However, this pattern does not necessarily translate into lower stress levels, as women in the teaching profession often face additional psychosocial demands, including emotional labour and caregiving responsibilities, which may offset the benefits of healthy dietary habits [36,60].

Participants with higher MD adherence were more likely to engage in elevated levels of physical activity, reflecting the well-documented clustering of health-promoting behaviours wherein individuals adopting one healthy lifestyle habit tend to engage in others [1,11]. The MD, often considered a proxy for an overall healthy lifestyle, has been consistently associated with additional positive behaviours, such as regular physical activity, particularly among individuals with higher health literacy, better access to health-related resources, and stronger intrinsic motivation [1,11]. Despite the observed associations, no significant relationship emerged between MD adherence and either perceived stress or sleep quality after adjustment. Although surprising, this finding aligns with the previous studies showing that the psychological benefits typically attributed to the MD were attenuated or lost after controlling for relevant covariates [61]. These findings suggest that, while MD adherence may generally improve psychological outcomes in the general population, its effects might be modulated by contextual factors, such as occupational stress.

Physical activity is a well-documented modulator of both sleep quality and psychological stress. Regular engagement in moderate activity has been shown to improve sleep architecture, to reduce sleep latency, and to enhance subjective sleep satisfaction [62]. In line with the existing research, we observed that perceived stress was inversely correlated with physical activity, suggesting that regular engagement in physical activity may serve as a protective factor against elevated stress levels [63,64,65]. Nevertheless, for the adjusted analyses, this association did not remain significant. One possible explanation is that sleep quality may function as a mediating or moderating variable in the relationship between stress and physical activity. Indeed, recent studies have suggested that the mental health benefits of physical activity are, in part, mediated through enhancements in sleep quality [6,66,67]. Moreover, the timing and intensity of physical activity appear to play a crucial role; for instance, high-intensity exercise performed in the late afternoon or evening may increase physiological arousal and interfere with circadian regulation, potentially impairing sleep onset and continuity [68].

A key and consistent finding of the present study was the strong bidirectional association between perceived stress and sleep quality. Poorer sleep predicted higher levels of perceived stress, while elevated stress was, in turn, associated with worse sleep outcomes. This reciprocal association is well supported by the literature and underscores a self-perpetuating cycle wherein psychological and physiological processes mutually reinforce each other [69,70]. Such dynamics are particularly relevant for high-stress occupational environments, like education, where sustained emotional demands, excessive workloads, and time and pressure frequently undermine well-being and job performance [21,38]. The present findings are consistent with research conducted in other European contexts, such as Spain [71] and Germany [72], where teachers similarly reported high levels of perceived stress and poor sleep quality, often associated with workload, emotional demands, and work–life imbalance. In Brazil, for instance, stress was found to increase the likelihood of poor sleep quality among teachers by 3.32 times, further reinforcing the consistency of this pattern across diverse cultural contexts [69,70]. Furthermore, the evidence from a study in China indicated that perceived stress mediates the relationship between poor sleep quality and internalizing symptoms, such as depression and anxiety [69,70]. These cross-national similarities suggest that stress and sleep disturbances are pervasive within this professional group, regardless of regional or dietary variations.

Neurobiological mechanisms may underpin this cycle. Sleep deprivation has been shown to impair prefrontal–limbic connectivity, reducing emotional regulation and increasing reactivity to stressors [73,74,75]. Conversely, chronic stress disrupts sleep architecture, prolongs latency, and reduces sleep efficiency, thereby amplifying emotional burden and reducing recovery capacity [75,76,77].

A counterintuitive yet noteworthy finding was the presence of high perceived stress levels among individuals who reported good subjective sleep quality. This apparent contradiction suggests a dissociation between how sleep is experienced and the underlying psychological state. One possible explanation is the phenomenon of sleep misperception, where individuals perceive their sleep as adequate despite ongoing emotional or cognitive arousal that is not resolved during rest. In such cases, stress may remain elevated due to unresolved mental processes, even in the absence of overt sleep complaints [78,79,80]. This has been observed in populations exposed to prolonged stress, such as healthcare workers during the COVID-19 pandemic, who maintained regular sleep routines while reporting persistent psychological strain [81]. Another interpretation is that individuals under high stress may intentionally adopt health-promoting behaviours, such as structured sleep routines or physical activity, to mitigate stress, thereby improving their sleep experience without necessarily reducing their stress perception. Alternatively, stress-induced hyperarousal may impair the perception of stress without fully disrupting sleep patterns [73]. These hypotheses highlight the importance of considering psychological resilience, individual differences, and contextual variables in interpreting stress–sleep dynamics.

Chronic exposure to high job demands, insufficient recovery time, and lack of institutional support contribute to fatigue, burnout, and unhealthy coping behaviours [23,82]. Moreover, organisational cultures that normalise overwork and underprioritise well-being may lead teachers to neglect basic health needs, including balanced diet, regular exercise, and restorative sleep [25,83]. These issues transcend individual responsibility, reflecting systemic conditions that shape behavioural patterns and health trajectories [84].

From a public health perspective, these findings reinforce the need for integrated, school-based health promotion programmes that simultaneously address multiple lifestyle factors. Interventions that combine sleep hygiene education, access to nutritious food options, and opportunities for physical activity are more likely to succeed than isolated, single-component strategies [33,85]. Moreover, organisational reforms are necessary to support these efforts, including policies to reduce excessive workload, to enhance job autonomy, and to promote work–life balance [86,87,88].

Promoting teacher well-being should be understood not only as an ethical obligation but as a strategic investment in educational effectiveness. Healthy, rested, and motivated teachers are more likely to engage students, to foster positive learning environments, and to contribute to school cohesion [35,37]. In this regard, this study supports a shift towards systemic, evidence-informed approaches that value teachers not only as educators but as individuals whose health directly influences the sustainability of the education system.

### 4.1. Practical Implications

The findings of this study highlight the need to develop tailored health promotion programmes within school settings, focusing specifically on teachers as key actors in the educational system. The observed associations between higher adherence to the Mediterranean Diet and physical activity, as well as the bidirectional relationship between sleep quality and perceived stress, support the implementation of integrated interventions that simultaneously address multiple lifestyle dimensions. In practical terms, schools and educational authorities could consider introducing initiatives such as workshops on nutrition and stress management, structured physical activity breaks during the workday, and awareness campaigns on sleep hygiene. Such measures have the potential to enhance teachers’ physical and mental well-being, to improve the overall school environment, and indirectly to contribute to teaching quality and educational outcomes.

### 4.2. Strengths, Limitations, and Future Directions

This study presents several strengths that reinforce its scientific relevance. It adopts a holistic and multidisciplinary perspective by analysing the interplay between dietary habits, sleep quality, physical activity, and perceived stress, rather than treating these factors in isolation. The inclusion of school teachers, a population often underrepresented in lifestyle and occupational health research [35,37], adds contextual value. Furthermore, the use of validated, internationally recognised instruments ensures the reliability and comparability of the findings [47,51,53,54]. This study also provides preliminary data that can inform the design of future interventions in educational settings.

However, several limitations must be acknowledged. Firstly, the cross-sectional design precludes causal inference, limiting the interpretation of the directionality between variables. Secondly, the small, non-probabilistic sample of 50 participants limits statistical power and generalisability. However, this sample size aligns with recommendations for exploratory pilot studies designed to assess feasibility and to identify preliminary behavioural patterns, rather than to test hypotheses formally [89,90]. Although the low proportion of male participants further restricts the potential for sex-based comparisons, the exploratory analyses revealed some differences between men and women in dietary and lifestyle patterns. These findings should be interpreted with caution and warrant further investigation in larger, more sex-balanced samples. Nevertheless, the diversity observed within the sample—regarding age, teaching level, and health indicators—contributes to the internal validity of the associations explored.

Thirdly, this study relied on self-reported data, which may be influenced by recall bias or social desirability. Although validated tools were used, objective measures (such as actigraphy or food diaries) would enhance accuracy in future studies [23,40]. The recruitment through digital platforms may also have introduced selection bias, favouring more health-conscious or digitally engaged individuals.

Moreover, relevant contextual and psychosocial variables, such as mental health status, work-related burnout, and family responsibilities, were not included but could have influenced the results. Their integration in future research would provide a more comprehensive understanding of the factors affecting teachers’ well-being [34,82].

Although conventional statistical methods were adequate for this exploratory phase, future studies would benefit from more advanced techniques. Mediation models could clarify indirect effects, such as the role of sleep in the relationship between diet and stress. Cluster or latent profile analysis may help identify behavioural typologies, while structural equation modelling could reveal the complex interactions among lifestyle factors [66,67,75]. These approaches would allow for a deeper exploration of the causal mechanisms and inform more targeted and effective interventions.

In light of these strengths and limitations, future research should prioritise larger and more representative samples, longitudinal or intervention-based designs, and the inclusion of additional contextual variables. These enhancements are essential to expand the evidence base and to support the development of tailored, evidence-informed health promotion strategies in school environments.

## 5. Conclusions

This exploratory study contributes to the understanding of lifestyle behaviours among primary and secondary school teachers in Portugal, highlighting the meaningful associations between Mediterranean Diet adherence, physical activity, sleep quality, and perceived stress. Our findings suggest that greater adherence to the Mediterranean Diet is associated with higher levels of physical activity, while perceived stress is closely linked to poorer sleep quality and reduced physical activity. These results underscore the interconnected nature of health-related behaviours and reinforce the need for integrated health promotion strategies targeting teachers. Such interventions may include workplace-based programmes focused on stress management, sleep hygiene, and dietary education, aiming to enhance teachers’ well-being and, ultimately, to support a healthier school environment. Further longitudinal and intervention-based studies are warranted to better understand the causal pathways and to evaluate the effectiveness of holistic health programmes tailored to the needs of educators.

## Figures and Tables

**Table 1:** Sociodemographic, professional, and lifestyle characteristics of the participants stratified by sex.

	All Population (n = 50)	Male (n = 9)	Female (n = 41)	*p*-Value
Age	53.0 (47.3–57.8)	55.0 (46.0–57.0)	52.0 (48.0–58.0)	0.695 ^a^
Residence Area, % (n)				
Metropolitan Area of Lisbon	68.0 (34)	66.7 (6)	68.3 (28)	
Centro Region	26.0 (13)	33.3 (3)	24.4 (10)	0.833 ^b^
Autonomous Region of the Madeira	6.0 (3)	0.0 (0)	7.3 (3)	
Educational Level Attended, % (n)				
Bachelor’s Degree	74.0 (37)	55.6 (5)	78.0 (32)	0.214 ^b^
Master’s/Doctorate	26.0 (13)	44.4 (4)	22.0 (9)	
Years of Teaching Experience	26.0 (21.3–32.8)	23.0 (20.0–30.0)	26.0 (22.0–34.0)	0.300 ^a^
Teaching Level, %				
Primary Education	10.0 (5)	22.2 (2)	7.3 (3)	
Upper Secondary Education	43.0 (21)	11.1 (1)	48.8 (20)	0.070 ^b^
High School Education	48.0 (24)	66.7 (6)	43.9 (18)	
Body Mass Index (n = 50)	23.1 (21.7–26.7)	26.1 (23.4–28.7)	23.1 (21.3–26.6)	0.065 ^a^
Nutritional Status, % (n)				
Underweight	2.0 (1)	0.0 (0)	2.4 (1)	
Normal weight	58.0 (29)	33.3 (3)	63.4 (26)	0.265 ^b^
Overweight	32.0 (16)	55.6 (5)	26.8 (11)	
Obese	8.0 (4)	11.1 (1)	7.3 (3)	
Smoking habits, % (n)				
Non-Smoker	76.0 (38)	66.7 (6)	78.1 (32)	0.668 ^b^
Smoker	24.0 (12)	33.3 (3)	22.0 (9)	

Data are presented as percentages (absolute values) or median (interquartile range) for categorical or continuous variables, respectively. Group comparisons were assessed using ^a^ Mann–Whitney U Test, ^b^ Fisher’s exact test, as appropriate.

**Table 2:** Mediterranean Diet adherence screener (MEDAS) components stratified by sex.

	Criteria for 1 Point	All Population (n = 50)	Male (n = 9)	Female (n = 41)	*p*-Value
1. Do you use olive oil as your main culinary fat?	Yes	98.0 (49)	88.9 (8)	100.0 (41)	0.180 ^a^
2. How much olive oil do you consume per day (including for frying, seasoning, salad dressing, meals eaten outside the home, etc.)?	≥4 Tablespoons	24.0 (12)	11.1 (1)	26.8 (11)	0.425 ^a^
3. How many servings of vegetables do you consume per day?	≥2	44.0 (22)	11.1 (1)	51.2 (21)	0.060 ^a^
4. How many pieces of fruit (including natural fruit juices) do you consume per day?	≥3	42.0 (21)	44.4 (4)	41.5 (17)	1.000 ^a^
5. How many servings of red meat, hamburgers, or meat products (ham, sausage, etc.) do you consume per day?	<1	64.0 (32)	33.3 (3)	70.7 (29)	0.055 ^a^
6. How many servings of butter, margarine, or cream do you consume per day?	<1	82.0 (41)	88.9 (8)	80.5 (33)	1.000 ^a^
7. How many sugary or carbonated beverages do you drink per day?	<1	94.0 (47)	100.0 (9)	68.3 (38)	1.000 ^a^
8. How many glasses of wine do you drink per week?	≥7 cups	18.0 (9)	33.3 (3)	14.6 (6)	0.334 ^a^
9. How many servings of pulses do you consume per week?	≥3	46.0 (23)	77.8 (7)	39.9 (16)	0.062 ^a^
10. How many servings of fish or seafood do you consume per week?	≥3	56.0 (28)	66.7 (6)	53.7 (22)	0.713 ^a^
11. How many times do you consume commercial bakery products or sweets (non-homemade), such as cakes, cookies, biscuits per week?	<3	70.0 (35)	88.9 (8)	65.9 (27)	0.247 ^a^
12. How many servings of nuts (walnuts, almonds, including peanuts) do you consume per week?)	≥3	46.0 (23)	33.3 (3)	48.8 (20)	0.479 ^a^
13. Do you preferentially consume chicken, turkey, or rabbit instead of beef, pork, hamburger, or sausage?	Yes	70.0 (35)	66.7 (6)	70.7 (29)	1.000 ^a^
14. How many times per week do you consume boiled vegetables, pasta, rice, or other dishes cooked with a sautéed base (tomato, onion, leek or garlic, and olive oil)?	≥2	90.0 (45)	66.7 (6)	95.1 (39)	0.035 ^a^
Global MEDAS score		9.0 (8.0–10.0)	9.0 (5.0–10.0)	9.0 (8.0–10.0)	0.887 ^b^

Data are presented as percentages (absolute values) or median (interquartile range). Group comparisons were assessed using ^a^ Fisher’s exact test or ^b^ Mann–Whitney U test, as appropriate.

**Table 3:** Correlation between Mediterranean Diet adherence, sleep quality, perceived stress, and physical activity.

	MEDAS Score	PSQI Score	PSS-10 Score
PSQI score	0.132 (*p* = 0.361)	-	-
PSS-10 score	−0.086 (*p* = 0.552)	0.346 (*p* = 0.014)	-
Physical activity levels	0.343 (*p* = 0.015)	0.168 (*p* = 0.242)	−0.297 (*p* = 0.036)

Group comparisons were assessed using Spearman’s rank correlation coefficient.

**Table 4:** Regression analyses examining associations among Mediterranean Diet adherence (MEDAS score), sleep quality (PSQI score), perceived stress (PSS-10 score), and physical activity levels.

	MEDAS Score		PSQI Score		PSS-10 Score		Physical Activity Levels	
	Coef. B (CI 95%)	*p*-Value	Coef. B (CI 95%)	*p*-Value	Coef. B (CI 95%)	*p*-Value	OR (CI 95%)	*p*-Value
MEDAS score	-		0.280 (−0.135; 0.419)	0.306	−0.282 (−0.368; 0.192)	0.529	0.452 (0.923; 2.67)	0.056
PSQI score	0.089 (−0.166; 0.517)	0.306	-		0.708 (0.154; 0.718)	0.003	0.080 (0.839; 1.40)	0.539
PSS-10 score	−0.034 (−0.451; 0.235)	0.529	0.267 (0.153; 0.712)	0.003	-		−0.077 (0.767; 1.120)	0.421
Physical Activity Levels								
High–Low	1.166 (−0.245; 1.510)	0.153	1.115 (−0.497; 1.111)	0.445	−1.882 (−1.127; 0.487)	0.429	-	

Coef. B = unstandardised regression coefficient; OR = odds ratio (for physical activity, categorical outcome); CI = confidence interval.

## Data Availability

Data are contained within the article.

## Supplementary Materials

The following supporting information can be downloaded at: https://www.mdpi.com/article/10.3390/nu17172745/s1, Table S1. Sociodemographic, professional, and lifestyle characteristics stratified by Mediterranean dietary pattern adherence. Table S2. Pittsburgh Sleep Quality Index Components Stratified by Mediterranean Diet Adherence. Table S3. Correlation between global sleep quality and PSQI Components. Table S4. Sociodemographic, professional, and lifestyle characteristics stratified by sleep quality. Table S5. Perceived Stress Scale—PSS-10 components stratified by Mediterranean diet adherence and sleep quality. Table S6. Sociodemographic, professional, and health-related characteristics stratified by perceived stress levels. Table S7. Sociodemographic, professional, and health-related characteristics stratified by physical activity levels.
