# Supplementary material for: Mediterranean Diet, Sleep Quality, Perceived Stress, and Physical Activity: A Pilot Study Among School Teachers
Source: Nutrients. 2025 Aug 25;17(17):2745. doi: 10.3390/nu17172745 (PMC12430172; doi:10.3390/nu17172745)
Supplement: Supplementary file 1 [file nutrients-17-02745-s001.zip › nutrients-3817726-supplementary.pdf]

nutrients

MDPI

Supplementary Materials

Questionário Bilingue / Bilingual Questionnaire (PT-EN)
Hábitos alimentares e estilos de vida em professores do ensino básico e secundário/
Eating habits and lifestyles among primary and secondary school teachers

Características sociodemográficas e de saúde/ Sociodemographic and health characteristics

Qual é a sua idade? (anos)
What is your age? (years)
Qual é o seu sexo?
What is your gender?
Qual é o seu estado civil?
What is your marital status?
Qual é a sua área de residência?
What is your region of residence?
Qual é o seu nível de educação mais elevado?
What is your highest educational level?
É professor de que nível de ensino?
At which educational level do you teach?
Qual é a sua área de formação base enquanto professor?
What is your main academic background as a teacher?
Há quantos anos exerce atividade como professor?
How many years have you been working as a teacher?
Qual é a sua carga horária semanal?
What is your weekly workload?
Reside longe do seu núcleo familiar?
Do you live far from your family?
Se sim, qual a distância aproximada (km)?
If yes, what is the approximate distance (km)?
Qual é o seu peso (kg)?
What is your weight (kg)?
Qual é a sua altura (m)?
What is your height (m)?
É fumador?
Do you smoke?
Se sim, que tipo de cigarros fuma?
If yes, what type of cigarettes do you smoke?
Quantos cigarros fuma por dia (em média)?
How many cigarettes do you smoke per day (on average)?

 https://doi.org/10.3390/nu17172745

Hábitos alimentares e adesão ao padrão alimentar mediterrâneo/ Eating habits and adherence to the Mediterranean dietary pattern

Como caracteriza o seu padrão alimentar?
How would you describe your eating pattern?
Utiliza o azeite como principal gordura culinária?
Do you use olive oil as your main culinary fat?
Que quantidade de azeite consome num dia?
How much olive oil do you consume per day?
Quantas porções de hortícolas consome por dia?
How many servings of vegetables do you consume per day?
Quantas peças de fruta consome por dia?
How many pieces of fruit do you consume per day?
Quantas porções de carne vermelha ou processada consome por dia?
How many servings of red or processed meat do you consume per day?
Quantas porções de manteiga, margarina ou natas consome por dia?
How many servings of butter, margarine or cream do you consume per day?
Qual a sua frequência de ingestão de refrigerantes?
What is your frequency of soft drink consumption?
Quantos copos de vinho bebe por semana?
How many glasses of wine do you drink per week?
Quantas porções de leguminosas consome por semana?
How many servings of legumes do you consume per week?
Quantas porções de peixe ou marisco consome por semana?
How many servings of fish or seafood do you consume per week?
Quantas vezes por semana consome produtos de pastelaria ou doces comerciais?
How often per week do you consume pastries or commercial sweets?
Quantas porções de oleaginosas consome por semana?
How many servings of nuts do you consume per week?
Consome preferencialmente frango, peru ou coelho em vez de outras carnes?
Do you prefer chicken, turkey or rabbit instead of other meats?
Quantas vezes por semana consome pratos confecionados com refogado?
How many times per week do you consume dishes cooked with sautéed vegetables?

Qualidade do sono/ Sleep quality

A que horas se deitou à noite na maioria das vezes (último mês)?
At what time did you usually go to bed at night (last month)?
Quanto tempo demorou para adormecer (média em minutos)?
How long did it take you to fall asleep (average in minutes)?
A que horas acordou na maioria das vezes (último mês)?
At what time did you usually wake up (last month)?
Quantas horas de sono por noite dormiu (último mês)?
How many hours of sleep per night did you have (last month)?
Durante o mês passado, quantas vezes teve problemas para dormir devido a diferentes fatores?
During the last month, how often did you have trouble sleeping due to various factors?

Como classificaria a qualidade do seu sono?
How would you rate your sleep quality?
Tomou algum medicamento para dormir no último mês?
Did you take any medication to help you sleep in the last month?
Teve problemas em ficar acordado durante atividades no último mês?
Did you have problems staying awake during activities in the last month?
Sentiu pouca vontade ou falta de entusiasmo para tarefas diárias?
Did you feel little desire or lack of enthusiasm for daily tasks?
Vive com um(a) companheiro(a)?
Do you live with a partner?
Se sim, pergunte-lhe se teve ressonar alto, pausas na respiração, movimentos de pernas ou episódios de confusão durante o sono
If yes, ask them if you had loud snoring, breathing pauses, leg movements or confusion episodes during sleep

Stress ocupacional/ Occupational stress
No último mês, com que frequência esteve preocupado por algo inesperado?
In the last month, how often have you been upset because of something unexpected?
No último mês, com que frequência se sentiu incapaz de controlar coisas importantes na sua vida?
In the last month, how often have you felt unable to control important things in your life?
No último mês, com que frequência se sentiu nervoso ou em stresse?
In the last month, how often have you felt nervous or stressed?
No último mês, com que frequência sentiu confiança para lidar com problemas pessoais?
In the last month, how often have you felt confident about handling personal problems?
No último mês, com que frequência sentiu que as coisas estavam a correr bem?
In the last month, how often have you felt that things were going your way?
No último mês, com que frequência sentiu que não conseguia lidar com tudo o que tinha para fazer?
In the last month, how often have you felt that you could not cope with all the things you had to do?
No último mês, com que frequência conseguiu controlar irritações?
In the last month, how often have you been able to control irritations?
No último mês, com que frequência sentiu ter tudo sob controlo?
In the last month, how often have you felt that you had everything under control?
No último mês, com que frequência ficou furioso por coisas fora do seu controlo?
In the last month, how often have you felt angered by things out of your control?
No último mês, com que frequência sentiu que as dificuldades se acumulavam a ponto de não conseguir superá-las?
In the last month, how often have you felt difficulties piling up so high that you could not overcome them?

Atividade física/ Physical activity
Nos últimos 7 dias, em quantos dias andou pelo menos 10 minutos seguidos?
In the last 7 days, on how many days did you walk for at least 10 continuous minutes?
Quanto tempo no total despendeu num desses dias a andar/caminhar (min)?
How much total time did you spend walking on one of those days (min)?
Num dia normal, quanto tempo passa sentado (horas)?
On a typical day, how much time do you spend sitting (hours)?

Nos últimos 7 dias, em quantos dias fez atividades físicas vigorosas?
In the last 7 days, on how many days did you do vigorous physical activities?
Nos dias em que pratica atividades vigorosas, quanto tempo dedica (min)?
On days when you do vigorous activities, how much time do you spend (min)?
Nos últimos 7 dias, em quantos dias fez atividades físicas moderadas?
In the last 7 days, on how many days did you do moderate physical activities?
Nos dias em que pratica atividades moderadas, quanto tempo dedica (min)?
On days when you do moderate activities, how much time do you spend (min)?
O que lhe dificulta ou impede a prática de atividade física?
What makes it difficult or prevents you from doing physical activity?

Table S1. Sociodemographic, professional, and lifestyle characteristics stratified by Mediterranean dietary pattern adherence.

	**Mediterranean Diet Adherence**			
	**Low (n=5)**	**Moderate (n=29)**	**High (n=16)**	*p-value*
**Age**	49.0 (46.0-49.0)	53.0 (48.0-58.0)	55.0 (46.0-57.5)	0.384[a]
**Sex,** % (n)				
Men	60.0 (3)	6.9 (2)	25.0 (4)	**0.012**[b]
Women	40.0 (2)	93.1 (27)	75.0 (12)	
**Residence Area**, % (n)				
Metropolitan Area of Lisbon	20.0 (1)	75.9 (22)	68.8 (11)	0.107[b]
Centro Region	60.0 (3)	20.7 (6)	25.0 (4)	
Autonomous Region of the Madeira	20.0 (1)	3.4 (1)	6.3 (1)	
**Educational Level Attended**, % (n)				
Bachelor's Degree	40.0 (2)	75.9 (22)	81.3 (13)	0.189[b]
Master / Doctorate	60.0 (3)	24.1 (7)	18.8 (3)	
**Years of Teaching Experience**	23.0 (17.0-26.0)	26.0 (22.0-34.0)	28.5 (21.5-30.5)	0.461[a]
**Teaching Level,** %				
Primary Education	20.0 (1)	6.9 (2)	12.5 (2)	0.435[b]
Upper Secondary Education-	40.0 (2)	51.7 (15)	31.3 (5)	
High School Education	60.0 (3)	41.4 (12)	56.3 (9)	
**Body Mass Index**	23.1 (22.9-23.1)	23.9 (21.8-27.3)	23.0 (21.1-26.8)	0.682[a]
**Nutritional Status**, % (n)				
Underweight	0.0 (0)	0.0 (0)	6.3 (1)	0.831[b]
Normal weight	80.0 (4)	55.2 (16)	56.3 (9)	
Overweight	20.0 (1)	34.5 (10)	31.2 (5)	
Obesity	0.0 (0)	10.3 (3)	6.3 (1)	
**Smoking habits**, % (n)				
Non-Smoker	80.0 (4)	72.4 (21)	81.3 (13)	0.883[b]
Smoker	20.0 (1)	27.6 (8)	18.8 (3)	

Data are presented as percentages (absolute values) or median (interquartile range) for categorical or continuous variables, respectively. Group comparisons were assessed using [a]Kruskal-Wallis test or [b]Fisher's Exact test, as appropriate.

Table S2. Pittsburgh Sleep Quality Index Components Stratified by Mediterranean Diet Adherence.

PSQI components	All population (n=50)	Mediterranean Diet Adherence			*p-value*
		Low (n=5)	Moderate (n=29)	High (n=16)	
**Subjective Sleep Quality**					
Very good	4.0 (2)	0.0 (0)	6.9 (2)	0.0 (0)	0.898[a]
Good	58.0 (29)	60.0 (3)	55.2 (16)	62.5 (10)	
Poor	34.0 (17)	40.0 (2)	31.0 (9)	37.6 (6)	
Very poor	4.0 (2)	0.0 (0)	6.9 (2)	0.0 (0)	
**Sleep Latency**					
≤ 15 min	38.0 (19)	80.0 (4)	34.5 (10)	31.3 (5)	0.711[a]
16 to 30 min	38.0 (19)	20.0 (1)	41.4 (12)	37.6 (6)	
31 to 60 min	18.0 (9)	0.0 (0)	17.2 (5)	25.0 (4)	
> 60 min	6.0 (3)	0.0 (0)	6.9 (2)	6.3 (1)	
**Sleep Duration**					
>7 h	24.0 (12)	20.0 (1)	27.6 (8)	18.8 (3)	0.717[a]
6 to 7 h	68.0 (34)	60.0 (3)	65.5 (19)	75.0 (12)	
5 to 6 h	6.0 (3)	20.0 (1)	3.4 (1)	6.3 (1)	
< 5 h	2.0 (1)	0.0 (0)	3.4 (1)	0.0 (0)	
**Sleep Efficiency**					
>85%	66.0 (33)	60.0 (3)	69.0 (20)	62.5 (10)	0.544[a]
75% to 84%	18.0 (9)	20.0 (1)	10.4 (3)	31.3 (5)	
65% a 74%	12.0 (6)	20.0 (1)	13.8 (4)	6.3 (1)	
<65%	4.0 (2)	0.0 (0)	6.9 (2)	0.0 (0)	
**Sleep Disturbances**					
0	4.0 (2)	0.0 (0)	3.4 (1)	6.3 (1)	0.956[a]
1 to 9	84.0 (42)	100.0 (5)	79.3 (23)	87.5 (14)	
10 to 18	10.0 (5)	0.0 (0)	13.8 (4)	6.3 (1)	
19 to 27	2.0 (1)	0.0 (0)	3.4 (1)	0.0 (0)	
**Sleep Medication**					
Never	66.0 (33)	80.0 (4)	69.0 (20)	56.3 (9)	0.764[a]
Less than once a week	8.0 (4)	20.0 (1)	6.9 (2)	6.3 (1)	
Once or twice a week	10.0 (5)	0.0 (0)	10.4 (3)	12.5 (2)	
Three or more times per week	16.0 (8)	0.0 (0)	13.8 (4)	25.0 (4)	
**Daytime Dysfunction**					
0	14.0 (7)	0.0 (0)	10.3 (3)	25.0 (4)	0.746[a]
1 and 2	64.0 (32)	80.0 (4)	62.1 (18)	62.5 (10)	
3 and 4	18.0 (9)	20.0 (1)	20.7 (6)	12.5 (2)	
5 and 6	4.0 (2)	0.0 (0)	6.9 (2)	0.0 (0)	
**Global PSQI score**	6.0 (4.0-7.8)	6.0 (44.0-7.0)	6.0 (4.0-8.0)	6.5 (4.8-7.3)	0.880[b]
**Sleep Quality**					
Good	60.0 (30)	60.0 (3)	62.1 (18)	56.3 (9)	0.912[a]
Poor	40.0 (20)	40.0 (2)	37.9 (11)	43.8 (7)	

Data are presented as percentages (absolute values). Group comparisons were assessed using [a]Fisher's Exact test.

Table S3. Correlation between global sleep quality and PSQI Components.

	Global PSQI score	
	**Correlation**	***p-value***
**Subjective Sleep Quality**	0.663	**<0.001**
**Sleep Latency**	0.609	**<0.001**
**Sleep Duration**	0.404	**0.004**
**Sleep Efficiency**	0.690	**<0.001**
**Sleep Disturbances**	0.569	**<0.001**
**Sleep Medication**	0.770	**<0.001**
**Daytime Dysfunction**	0.429	**0.002**

Group comparisons were assessed using Spearman's rank-order correlation coefficient.

Table S4. Sociodemographic, professional, and lifestyle characteristics stratified by sleep quality.

	Sleep Quality		*p-value*
	**Poor (n=20)**	**Good (n=30)**	
**Age**	50.0 (47.8-56.0)	52.5 (46.5-57.8)	0.648[a]
**Sex,** % (n)			
Male	15.0 (3)	20.0 (6)	0.724[b]
Female	85.0 (17)	80.0 (24)	
**Residence Area**, % (n)			
Metropolitan Area of Lisbon	65.0 (13)	70.0 (21)	0.700[b]
Centro Region	25.0 (5)	26.7 (8)	
Autonomous Region of the Madeira	10.0 (2)	3.3 (1)	
**Educational Level Attended**, % (n)			
Bachelor's Degree	80.0 (16)	70.0 (21)	0.522[b]
Master / Doctorate	20.0 (4)	30.0 (9)	
**Years of Teaching Experience**	24.5 (20.0-28.5)	29.5 (22.3-34.8)	0.104[a]
**Teaching Level,** %			
Primary Education	0.0 (0)	16.7 (5)	0.198[b]
Upper Secondary Education	45.0 (9)	40.0 (12)	
High School Education	36.7 (11)	43.3 (13)	
**Body Mass Index**	24.9 (4.1)	24.1 (3.8)	0.497[c]
**Nutritional Status**, % (n)			
Underweight	0.0 (0)	3.3 (1)	0.391[b]
Normal weight	60.0 (12)	56.7 (17)	
Overweight	25.0 (5)	36.7 (11)	
Obesity	15.0 (3)	3.3 (1)	
**Smoking habits**, % (n)			
Non-Smoker	80.0 (16)	73.3 (22)	0.740[b]
Smoker	20.0 (4)	26.7 (8)	

Data are presented as percentages (absolute values) or median (interquartile range) for categorical or continuous variables, respectively. Group comparisons were assessed using [a]Mann-Whitney U Test, [b]Student t Test, [c]Chi-squared test, or [d]Fisher's Exact test, as appropriate.

Table S5. Perceived Stress Scale - PSS-10 components stratified by Mediterranean diet adherence and sleep quality.

PSS components	All population (n=50)	Mediterranean Diet Adherence			*p-value*	Sleep Quality		*p-value*
		Low (n=5)	Moderate (n=29)	High (n=16)		Poor (n=20)	Good (n=30)	
**Been upset because something unexpected happened**								
Never	8.0 (4)	0.0 (0)	10.3 (3)	6.3 (1)		20.0 (4)	0.0 (0)	
Almost never	20.0 (10)	40.0 (2)	13.8 (4)	25.0 (4)		30.0 (6)	13.3 (4)	
Sometimes	52.0 (26)	60.0 (3)	44.8 (13)	62.5 (10)	0.598[a]	50.0 (10)	53.3 (16)	**0.004**[a]
Infrequently	12.0 (6)	0.0 (0)	17.2 (5)	6.3 (1)		0.0 (0)	20.0 (6)	
Very frequently	8.0 (4)	0.0 (0)	13.8 (4)	0.0 (0)		0.0 (0)	13.3 (4)	
**Felt unable to control important things in your life**								
Never	24.0 (12)	20.0 (1)	17.2 (5)	37.5 (6)		35.0 (7)	16.7 (5)	
Almost never	40.0 (20)	60.0 (3)	41.4 (12)	31.3 (5)		40.0 (8)	40.0 (12)	
Sometimes	22.0 (11)	20.0 (1)	17.2 (5)	31.3 (5)	0.433[a]	25.0 (5)	20.0 (6)	0.182[a]
Infrequently	10.0 (5)	0.0 (0)	17.2 (5)	0.0 (0)		0.0 (0)	16.7 (5)	
Very frequently	4.0 (2)	0.0 (0)	6.9 (2)	0.0 (0)		0.0 (0)	6.7 (2)	
**Felt nervous or stressed**								
Never	6.0 (3)	0.0 (0)	6.9 (2)	6.3 (1)		15.0 (3)	0.0 (0)	
Almost never	28.0 (14)	40.0 (2)	24.1 (7)	31.3 (5)		40.0 (8)	20.0 (6)	
Sometimes	50.0 (25)	40.0 (2)	51.7 (15)	50.0 (8)	0.883[a]	45.0 (9)	53.3 (16)	**0.016**[a]
Infrequently	10.0 (5)	20.0 (1)	6.9 (2)	12.5 (2)		0.0 (0)	16.7 (5)	
Very frequently	6.0 (3)	0.0 (0)	10.3 (3)	0.0 (0)		0.0 (0)	10.0 (3)	
**Felt confident about your ability to handle personal problems**								
Never	12.0 (6)	20.0 (1)	6.9 (2)	18.8 (3)		15.0 (3)	10.0 (3)	
Almost never	22.0 (11)	0.0 (0)	20.7 (6)	31.3 (5)		30.0 (6)	16.7 (5)	
Sometimes	44.0 (22)	80.0 (4)	48.3 (14)	25.0 (4)	0.422[a]	25.0 (5)	56.7 (17)	0.128[a]
Infrequently	18.0 (9)	0.0 (0)	20.7 (6)	18.8 (3)		20.0 (4)	16.7 (5)	
Very frequently	4.0 (2)	0.0 (0)	3.4 (1)	6.3 (1)		10.0 (2)	0.0 (0)	
**Felt that things were going your way**								
Never	4.0 (2)	0.0 (0)	31.0 (9)	12.5 (2)		0.0 (0)	6.7 (2)	
Almost never	26.0 (13)	20.0 (1)	27.6 (8)	25.0 (4)		30.0 (6)	23.3 (7)	
Sometimes	36.0 (18)	40.0 (2)	31.0 (9)	43.8 (7)	0.281[a]	45.0 (9)	30.0 (9)	0.280[a]
Infrequently	28.0 (14)	20.0 (1)	37.9 (11)	12.5 (2)		15.0 (3)	36.7 (11)	
Very frequently	6.0 (3)	20.0 (1)	3.4 (1)	6.3 (1)		10.0 (2)	3.3 (1)	
**Found that you could not cope with all the things you had to do**								
Never	20.0 (10)	40.0 (2)	10.3 (3)	31.3 (5)	0.547[a]	25.0 (5)	16.7 (5)	0.207[a]
Almost never	28.0 (14)	40.0 (2)	31.0 (9)	18.8 (3)		25.0 (5)	30.0 (9)	

Sometimes	32.0 (16)	20.0 (1)	31.0 (9)	37.5 (6)		45.0 (9)	23.3 (7)	
Infrequently	16.0 (8)	0.0 (0)	20.7 (6)	12.5 (2)		5.0 (1)	23.3 (7)	
Very frequently	4.0 (2)	0.0 (0)	6.9 (2)	0.0 (0)		0.0 (0)	6.7 (2)	
**Been able to control irritations in your life**								
Never	8.0 (4)	20.0 (1)	6.9 (2)	6.3 (1)		5.0 (1)	10.0 (3)	
Almost never	26.0 (13)	0.0 (0)	24.1 (7)	37.5 (6)		20.0 (4)	30.0 (9)	
Sometimes	54.0 (27)	80.0 (4)	55.2 (16)	43.8 (7)	0.733[a]	60.0 (12)	50.0 (15)	0.440[a]
Infrequently	8.0 (4)	0.0 (0)	10.3 (3)	6.3 (1)		5.0 (1)	10.0 (3)	
Very frequently	4.0 (2)	0.0 (0)	3.4 (1)	6.3 (1)		10.0 (2)	0.0 (0)	
**Felt that you were on top of things**								
Never	6.0 (3)	20.0 (1)	3.4 (1)	6.3 (1)		5.0 (1)	6.7 (2)	
Almost never	24.0 (12)	0.0 (0)	24.1 (7)	31.3 (5)		30.0 (6)	20.0 (6)	
Sometimes	36.0 (18)	60.0 (3)	27.6 (8)	43.8 (7)	0.417[a]	30.0 (6)	40.0 (12)	0.797[a]
Infrequently	28.0 (14)	20.0 (1)	34.5 (10)	18.8 (3)		25.0 (5)	30.0 (9)	
Very frequently	6.0 (3)	0.0 (0)	10.3 (3)	0.0 (0)		10.0 (2)	3.3 (1)	
**Been angered because things were out of your control**								
Never	12.0 (6)	0.0 (0)	13.8 (4)	12.5 (2)		15.0 (3)	10.0 (3)	
Almost never	60.0 (30)	40.0 (2)	55.2 (16)	75.0 (12)		70.0 (14)	53.3 (16)	
Sometimes	20.0 (10)	60.0 (3)	20.7 (6)	6.3 (1)	0.330[a]	15.0 (3)	23.3 (7)	0.300[a]
Infrequently	8.0 (4)	0.0 (0)	10.3 (3)	6.3 (1)		0.0 (0)	13.3 (4)	
Very frequently	0.0 (0)	0.0 (0)	0.0 (0)	0.0 (0)		0.0 (0)	0.0 (0)	
**Felt difficulties were piling up so high that you could not overcome them**								
Never	30.0 (15)	60.0 (3)	27.6 (8)	25.0 (4)		45.0 (9)	20.0 (6)	
Almost never	46.0 (23)	0.0 (0)	44.8 (13)	62.5 (10)		45.0 (9)	46.7 (14)	
Sometimes	16.0 (8)	40.0 (2)	17.2 (5)	6.3 (1)	0.249[a]	10.0 (2)	20.0 (6)	0.233[a]
Infrequently	6.0 (3)	0.0 (0)	6.9 (2)	6.3 (1)		0.0 (0)	10.0 (3)	
Very frequently	2.0 (1)	0.0 (0)	3.4 (1)	0.0 (0)		0.0 (0)	3.3 (1)	
**Global PSS-10 score**	16.0 (13.0-20.0)	15.0 (13.0-19.0)	17.0 (14.0-21.0)	14.5 (11.0-18.0)	0.228[b]	-	-	-
	16.5 (5.89)	-	-	-		14.3 (3.80)	18.0 (6.59)	**0.015**[c]

Data are presented as percentages (absolute values) or median (interquartile range). Group comparisons were assessed using [a]Fisher's Exact test, [b]Kruskal-Wallis test or [c]Welch test, as appropriate.

Table S6. Sociodemographic, professional, and health-related characteristics stratified by perceived stress levels.

	**Perceived Stress**			
	**Low (n=15)**	**Moderate (n=31)**	**High (n=4)**	*p-value*
**Age**	55.0 (48.5-56.5)	52.0 (47.5-57.5)	50.0 (38.3-58.3)	0.791[a]
**Sex,** % (n)				
Male	33.3 (5)	9.7 (3)	25.0 (1)	0.097[b]
Female	66.7 (10)	90.3 (28)	75.0 (3)	
**Residence Area**, % (n)				
Metropolitan Area of Lisbon	66.7 (10)	67.7 (21)	75.0 (3)	0.095[b]
Centro Region	20.0 (3)	32.3 (10)	0.0 (0)	
Autonomous Region of the Madeira	13.3 (2)	0.0 (0)	25.0 (1)	
**Educational Level Attended**, % (n)				
Bachelor's Degree	66.7 (10)	80.6 (25)	50.0 (2)	0.270[b]
Master / Doctorate	33.3 (5)	19.4 (6)	50.0 (2)	
**Years of Teaching Experience**	27.9 (6.3)	24.9 (9.6)	22.8 (14.8)	0.453[c]
**Teaching Level,** %				
Primary Education	6.7 (1)	12.9 (4)	0.0 (0)	0.588[b]
Upper Secondary Education	33.3 (5)	48.4 (15)	25.0 (1)	
High School Education	60.0 (9)	38.7 (12)	75.0 (3)	
**Body Mass Index**	25.3 (3.8)	23.9 (4.0)	24.5 (3.1)	0.560[c]
**Nutritional Status**, % (n)				
Underweight	0.0 (0)	3.3 (1)	0.0 (0)	0.862[b]
Normal weight	46.7 (7)	61.3 (19)	75.0 (3)	
Overweight	40.0 (6)	29.0 (9)	25.0 (1)	
Obesity	13.3 (2)	6.5 (2)	0.0 (0)	
**Smoking habits**, % (n)				
Non-Smoker	93.3 (14)	71.0 (22)	50.0 (2)	0.098[b]
Smoker	6.7 (1)	29.0 (9)	50.0 (2)	
**MEDAS score**	9.0 (8.0-10.0)	9.0 (6.5-10.0)	8.5 (8.0-9.0)	0.812[a]
**Mediterranean Diet Adherence, % (n)**				
Low	13.3 (2)	9.7 (3)	0.0 (0)	0.317[b]
Moderate	40.0 (6)	61.3 (19)	100.0 (4)	
High	46.7 (7)	29.0 (9)	0.0 (0)	
**PSQI score**	5.0 (3.5-7.0)	6.0 (4.5-7.5)	8.5 (7.5-11.5)	**0.043**[a]
**Sleep quality, % (n)**				
Poor	60.0 (9)	35.5 (11)	0.0 (0)	0.069[b]
Good	40.0 (6)	64.5 (20)	100.0 (4)	

Data are presented as median (interquartile range) or percentages (absolute values) for continuous or categorical variables, respectively. Group comparisons were assessed using [a]Kruskal-Wallis test [b]Fisher's Exact test or [c]ANOVA test, as appropriate.

Table S7. Sociodemographic, professional, and health-related characteristics stratified by physical activity levels.

	Physical Activity Levels			*p-value*
	**Low (n=9)**	**Moderate (n=19)**	**High (n=22)**	
**Age**	45.2 (11.7)	52.5 (6.1)	54.1 (6.6)	0.133[a]
**Sex,** % (n)				
Male	0.0 (0)	15.8 (3)	27.3 (6)	0.209[b]
Female	100.0 (9)	84.2 (16)	72.7 (16)	
**Residence Area**, % (n)				
Metropolitan Area of Lisbon	55.6 (5)	57.9 (11)	81.8 (18)	
Centro Region	44.4 (4)	31.6 (6)	13.6 (3)	0.281[b]
Autonomous Region of the Madeira	0.0 (0)	10.5 (2)	4.5 (1)	
**Educational Level Attended**, % (n)				
Bachelor's Degree	44.4 (4)	84.2 (16)	77.3 (17)	0.079[b]
Master / Doctorate	55.6 (5)	15.8 (3)	22.7 (5)	
**Years of Teaching Experience**	26.0 (17.0-26.0)	26.0 (21.0-32.0)	29.5 (23.0-35.0)	0.200[c]
**Teaching Level,** %				
Primary Education	0.0 (0)	21.1 (4)	4.5 (1)	
Upper Secondary Education	55.6 (5)	42.1 (8)	36.4 (8)	0.327[b]
High School Education	44.4 (4)	36.8 (7)	59.1 (13)	
**Body Mass Index**	26.7 (4.7)	23.4 (3.7)	24.3 (3.4)	0.228[a]
**Nutritional Status**, % (n)				
Underweight	0.0 (0)	0.0 (0)	4.5 (1)	
Normal weight	44.4 (4)	73.7 (14)	50.0 (11)	0.323[b]
Overweight	33.3 (3)	21.1 (4)	40.9 (9)	
Obesity	22.3 (2)	5.3 (1)	4.5 (1)	
**Smoking habits**, % (n)				
Non-Smoker	77.8 (7)	78.9 (15)	72.7 (16)	0.909[b]
Smoker	22.3 (2)	21.1 (4)	27.3 (6)	
**MEDAS score**	8.0 (6.0-9.0)	9.0 (8.0-9.0)	9.5 (8.3-10.0)	0.056[c]
**Mediterranean Diet Adherence, % (n)**				
Low	11.1 (1)	10.5 (2)	9.1 (2)	
Moderate	77.8 (7)	68.4 (13)	40.9 (9)	0.170[b]
High	11.1 (1)	21.1 (4)	50.0 (11)	
**PSQI score**	6.0 (3.0-7.0)	6.0 (4.0-7.5)	7.0 (5.0-7.6)	0.496[c]
**Sleep quality,** % (n)				
Poor	44.4 (4)	42.1 (8)	36.4 (8)	0.866[b]
Good	55.6 (5)	57.9 (11)	63.6 (14)	
**PSS-10 score**	18.7 (5.2)	17.6 (6.1)	14.7 (5.7)	0.149[a]
**Stress levels,** % (n)				
Low	11.1 (1)	31.6 (6)	36.4 (8)	
Moderate	77.8 (7)	57.9 (11)	59.1 (13)	0.637[b]
High	11.1 (1)	10.5 (2)	4.5 (1)	

Data are presented as median (interquartile range) or percentages (absolute values) for continuous or categorical variables, respectively. Group comparisons were assessed using [a]ANOVA test, [b]Fisher's Exact test or [c]Kruskal-Wallis test, as appropriate.
